# Association between *P^16INK4a^* Promoter Methylation and Non-Small Cell Lung Cancer: A Meta-Analysis

**DOI:** 10.1371/journal.pone.0060107

**Published:** 2013-04-05

**Authors:** Jundong Gu, Yanjun Wen, Siwei Zhu, Feng Hua, Hui Zhao, Hongrui Xu, Jiacong You, Linlin Sun, Weiqiang Wang, Jun Chen, Qinghua Zhou

**Affiliations:** 1 Tianjin Key Laboratory of Lung Cancer Metastasis and Tumor Microenvironment, Tianjin Lung Cancer Institute, Tianjin Medical University General Hospital, Tianjin, China; 2 Department of Neuropathology, Neurology Institute, Tianjin Medical University General Hospital, Tianjin, China; 3 Department of Oncology, Tianjin Union Medical Center, Tianjin, China; 4 Department of Thoracic Surgery, Shandong Cancer Hospital, Shandong, China; 5 Department of Thoracic Surgery, Tianjin Union Medical Center, Tianjin, China; University of Nebraska Medical Center, United States of America

## Abstract

**Background:**

Aberrant methylation of CpG islands acquired in tumor cells in promoter regions plays an important role in carcinogenesis. Accumulated evidence demonstrates *P^16INK4a^* gene promoter hypermethylation is involved in non-small cell lung carcinoma (NSCLC), indicating it may be a potential biomarker for this disease. The aim of this study is to evaluate the frequency of *P^16INK4a^* gene promoter methylation between cancer tissue and autologous controls by summarizing published studies.

**Methods:**

By searching Medline, EMBSE and CNKI databases, the open published studies about *P^16INK4a^* gene promoter methylation and NSCLC were identified using a systematic search strategy. The pooled odds of *P^16INK4A^* promoter methylation in lung cancer tissue versus autologous controls were calculated by meta-analysis method.

**Results:**

Thirty-four studies, including 2 652 NSCLC patients with 5 175 samples were included in this meta-analysis. Generally, the frequency of *P^16INK4A^* promoter methylation ranged from 17% to 80% (median 44%) in the lung cancer tissue and 0 to 80% (median 15%) in the autologous controls, which indicated the methylation frequency in cancer tissue was much higher than that in autologous samples. We also find a strong and significant correlation between tumor tissue and autologous controls of *P^16INK4A^* promoter methylation frequency across studies (Correlation coefficient 0.71, 95% CI:0.51–0.83, *P*<0.0001). And the pooled odds ratio of *P^16INK4A^* promoter methylation in cancer tissue was 3.45 (95% CI: 2.63–4.54) compared to controls under random-effect model.

**Conclusion:**

Frequency of *P^16INK4a^* promoter methylation in cancer tissue was much higher than that in autologous controls, indicating promoter methylation plays an important role in carcinogenesis of the NSCLC. Strong and significant correlation between tumor tissue and autologous samples of *P^16INK4A^* promoter methylation demonstrated a promising biomarker for NSCLC.

## Introduction

Lung cancer, accounting for 13% (1.6 million) of the total cases and 18% (1.4 million) of the deaths, was the most commonly diagnosed cancer as well as the leading cause of cancer death worldwide in 2008 [1]. Benefiting from the tobacco control, lung cancer death rate is decreasing in western developed countries. However, it is increasing in developing countries such as China, where smoking prevalence is still increasing [1]. Non-small cell lung cancer, accounting for 80% of primary lung carcinomas, was the most common type with a 5-year survival rate ranging from 2 to 47% for different clinical stages and histopathology [2]. About twenty percent of NSCLC patients are suitable for surgery at the time of diagnosis, and the other 80%, receiving conventional chemoradiation, can only survive a short period of time [3]. Therefore, the early diagnosis is essential to the prolonged survival of this disease.

Tumor suppressor gene promoter methylation is considered as an important mechanism for its inactivation, which occurs in the early stage of the tumorigenesis for many types of cancer [2], [4]. Thus, detection of aberrant methylation of tumor suppressor genes could be a potential method for the early diagnosis of various types of cancer, including NSCLC. The aberrant methylation status of primary tumors can be detected by methylation specific PCR(MSP), which could detect one methylated allele in the presence of 10^3^–10^4^ unmethylated alleles [5]. And many studies have also shown that cancer-specific methylation of tumor suppressor genes can be found in autologous clinical samples such as plasma, serum, sputum or bronchoalveolar lavage fluid(BALF) of NSCLC, indicating that it can be potential biomarkers for non-invasive diagnosis of this disease [6]–[8]. But the frequency of DNA methylation in tumor suppressor genes between cancer tissue and autologous clinical samples ranged a lot among the published studies with small sample size. Accordingly, we performed a meta-analysis on the basis of published articles of *P^16INK4a^* promoter methylation and lung cancer in order to better identify the correlation of methylation status between cancer tissue and autologous samples.

## Materials and Methods

### Studies Identification

**Figure 1:**
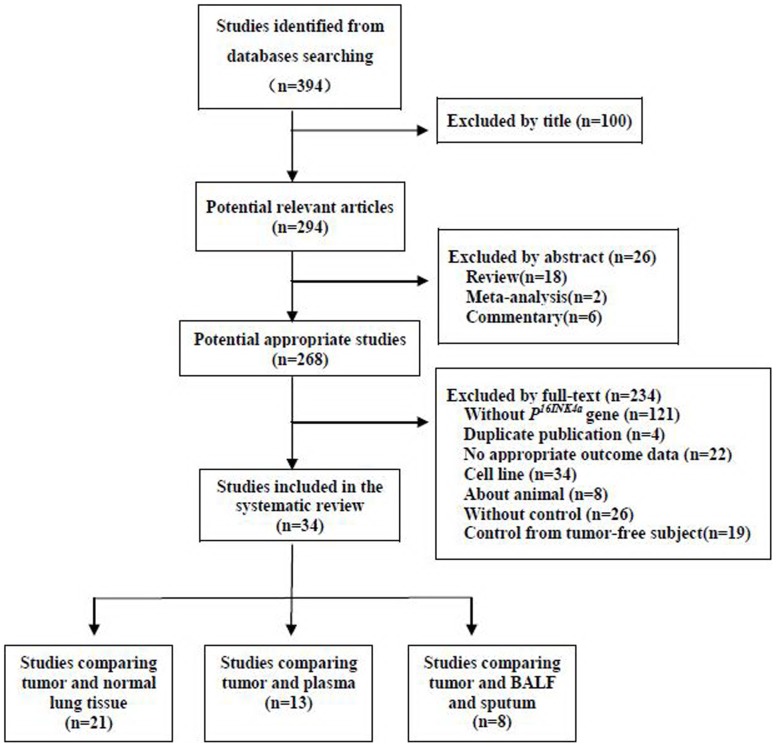
PRISMA flowchart of the literature search strategy for systematic review. (Data from some studies was used more than once, as they reported data in multiple controls.).

The selection procedure of studies was illustrated in the PRISMA (Preferred Reporting Items for Systematic Reviews and Meta-Analyses) statement flow chart (Fig. 1). Studies about *P^16INK4a^* gene promoter methylation in NSCLC, published before January 2012, were identified through an electronic sensitive search of Medline, EMBSE and CNKI databases. The searching strategy was performed using “Non-Small-Cell Lung Carcinoma” AND “methylation” as the Medical Subject Headings (MeSH) and corresponding free text word searching term. The title and abstract of initial identified articles were evaluated for appropriateness to the inclusion criteria. Then all potentially relevant articles were assessed in full-text paper and all references of included articles were further scanned for additional analysis.

### Data Extraction and Quality Assessment

The inclusion criteria of the meta-analysis was as follows: the patients were limited to non-small cell lung carcinoma without restriction of stages. The methods used for methylation detection were confined to methylation-specific polymerase chain reaction(MSP), real-time MSP(RT-MSP) and quantitative MSP(q-MSP). The results were the *P^16INK4A^* gene promoter methylation status in tumor tissue and corresponding autologous controls, including non-tumor lung tissue(NLT), plasma, sputum and bronchoalveolar lavage fluid(BALF) of NSCLC patients. Information on the name of the first author, year of publication, region of the included subjects and methylation status of *P^16INK4A^* gene in cancer tissue and controls were recorded from each study. Detailed information about each article was extracted by two reviewers (JG and YW) and then checked by the third reviewer (SZ) as described in the Cochrane Handbook for systematic reviews [9].

### Statistical Analysis

STATA/SE 11.0 (StataCorp LP, http://www.stata.com) and MetaAanlyst 3.13 (http://www.biomedcentral.com) were used for statistical analysis. Methylation status in tumor tissue and controls was calculated as methylation rate. The odds of *P^16INK4A^* promoter methylation in lung cancer tissue versus autologous controls was expressed as the odds ratio (OR) and its 95% confidence intervals (CI). Statistical heterogeneity across studies was assessed by chi-square (χ^2^) test [10], and the inconsistency was calculated by I^2^ [11]. If heterogeneity was significant (χ^2^, *p*<0.1 or I^2^>50%), meta-regression analysis was employed for further evaluation of the source of heterogeneity. And subgroup analysis according to the source of heterogeneity was performed for further evaluation. Finally, if significant heterogeneity across studies was detected and no appropriate clinical explanation of the heterogeneity was found, the random-effect method (Dersimonian-Laird method) was used to pool the data. Inversely, without significant heterogeneity between studies, fixed-effect method was purchased. And sensitivity analysis was also performed to assess the contribution of each study to the final results of the meta-analysis. The Begger’s funnel plot and Egger’s test were used to evaluate the possible publication bias [12]. The correlation of *P^16INK4A^* gene promoter methylation between tumor tissue and autologous clinical control samples were compared by Spearman’s rank correlation test.

## Results

### Study Characteristics

A total of three hundred and ninety-four studies were initially identified by searching the electronic databases. And 268 potential applicable articles, published from 2000 and 2012, were retrieved in full-text. Of those, 234 studies were excluded for the reasons: about other genes methylation status without *P^16INK4A^*, duplicated publication, no appropriate outcome data, about cell lines, about animals, without proper controls. Finally, thirty-four studies [6]–[8], [13]–[43] that reported data of methylation frequency in non-small-cell lung carcinoma tissue, and autologous controls were finally pooled in the meta-analysis (Fig. 1). Of the 34 included articles, 25 were conducted in Asia-Pacific(18 in Chinese mainland, 3 in Taiwan, 1 in Hong Kong, 2 in Korea, 1 in Japan), 4 in USA and 5 in Europe (3 in Italy, 1 in Greece, 1in England). Some of the included studies reported methylation status separately according to gender, histopathology types, smoking status and tumor stages. The general characteristics of included studies were summarized in table 1.

**Table 1:** General characteristics of included studies.

					Sample size (n)			Histology			Control type
Author	Year publication	Location	Age(y)	Gender(M/F)	T	C	Method	Sq	Ad	Ots	
Seike [13]	2000	Japan	63.7(40–80)	15/6	21	21	MSP	9	12	0	NLT
Su [16]	2000	China	58.9	Na	72	10	MSP	39	31	2	NLT
He [31]	2001	China	Na	Na	30	30	MSP	17	11	2	NLT
Zochbauer [6]	2001	USA	Na	76/31	107	104	MSP	43	45	19	NLT
Bearzatto [30]	2002	Italy	64	28/7	35	35	RT-MSP	10	18	7	Plasm
Chen [25]	2002	Taiwan	Na	Na	67	21	MSP	Na	Na	Na	Sputum
He [32]	2002	China	Na	Na	21	21	MSP	12	9	0	BALF
Ng [17]	2002	Hong Kong	60.2	25/8	33	33	MSP	13	15	5	Plasm,BALF
Cai [33]	2003	China	59.5	46/23	69	69	MSP	25	36	8	Plasm
Harden [26]	2003	USA	67(40–87)	50/40	90	90	q-MSP	33	36	21	NLT
Liu [18]	2003	China	Na	Na	98	110	MSP	58	40	0	Plasm sputum
Guo [27]	2004	USA	66.1(42–83)	Na	20	20	MSP	1	18	1	NLT BLAF
Liu [14]	2004	China	Na	Na	40	40	MSP	23	17	0	Plasm
Zhang [34]	2004	China	Na	Na	40	40	MSP	23	14	3	NLT
Russo [19]	2005	USA	Na	Na	48	48	MSP	Na	Na	Na	Plasm
Georgiou [23]	2007	Greece	63(38–76)	32/3	35	35	MSP	15	17	3	NLT BALF
Li [35]	2006	China	Na	38/11	49	49	MSP	22	24	3	Plasm
Rosalia [29]	2006	Italy	60.2(51–74)	20/9	29	18	MSP	5	23	1	Sputum
Ulivi [15]	2006	Italy	Na	49/12	61	61	RT-MSP	16	36	9	Plasm
Wang [36]	2006	China	32–73	42/5	47	47	MSP	31	7	9	NLT
Belinsky [20]	2007	England	62(37–80)	49/23	72	72	MSP	22	29	21	Plasm Sputum
Hong [22]	2007	Korea	Na	63/18	81	81	RT-MSP	40	34	7	NLT
Hsu(1) [21]	2007	Taiwan	69	45/18	63	63	q-MSP	41	13	9	NLT Plasm
Hsu(2) [24]	2007	Taiwan	Na	Na	82	82	MSP	37	23	22	NLTSputum
Kim [28]	2007	Korea	63±8.4	80/19	99	99	MSP	61	38	0	NLT
Yang [37]	2007	China	56(31–77)	34/15	49	49	MSP	26	23	0	NLT
Zhang [38]	2007	China	Na	Na	29	29	MSP	7	16	6	NLT
Guo [39]	2008	China	59±13	72/34	106	106	MSP	41	27	39	Plasm
Wang [8]	2008	China	Na	17/11	28	18	MSP	7	15	6	NLT
Chen [40]	2010	China	59.7(32–79)	102/18	120	120	MSP	66	26	28	NLT Plasm
Guo [41]	2010	China	59.2	23/5	28	28	MSP	Na	Na	Na	NLT
Zhang [42]	2006	China	52.3(37–73)	33/15	48	48	MSP	25	20	3	NLT
Zhang [7]	2011	China	61(32–79)	162/38	200	200	MSP	104	59	37	NLT
Sun [43]	2012	China	65	96/24	120	120	MSP	32	72	16	Sputum

M = male; F = female; T = tumor; C = control; Sq = squamous cell carcinoma; Ad = adenocarcinoma; Ots = others; BALF = bronchoalveolar lavage; NLT:non-tumor lung issue; Na = not available.

### Pooled Results from the Meta-analysis

**Figure 2:**
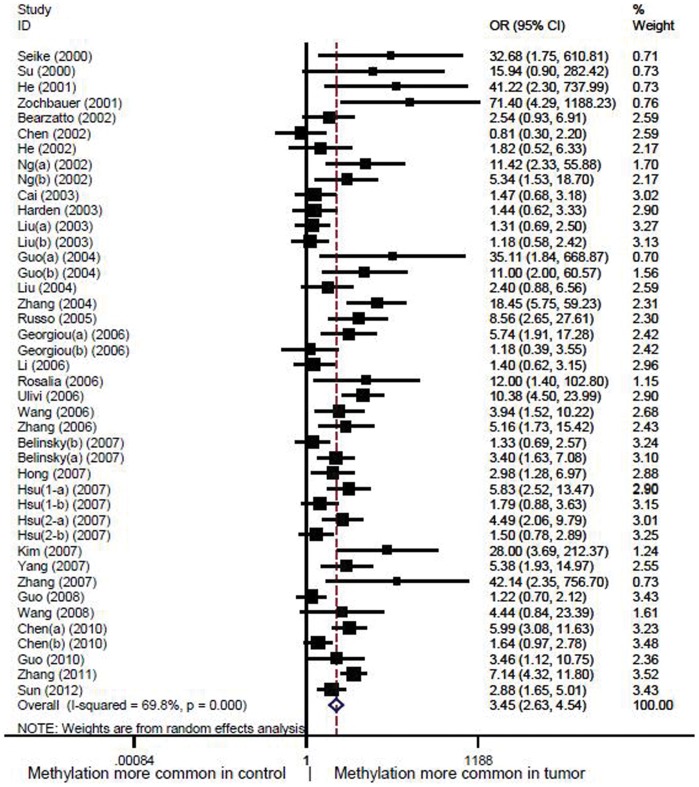
Forest plot of *P^16INK4A^* promoter methylation in cancer tissue versus autologous controls. The squares and horizontal lines represent the study-specific OR and 95% CI. The area of the squares reflects the weight (inverse of the variance). The diamond represents the pooled OR and 95% CI.

**Figure 3:**
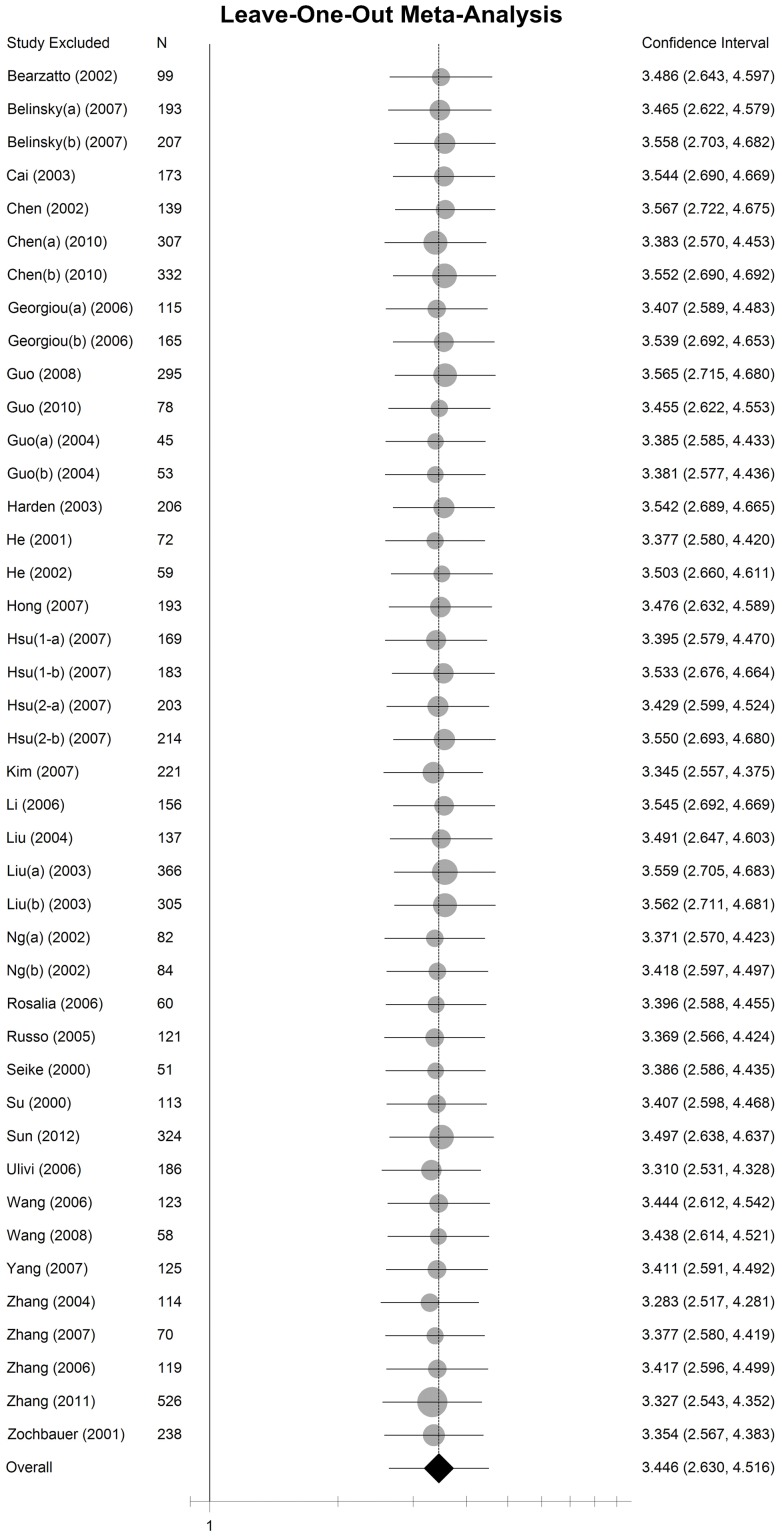
The sensitivity analysis by omitting a single study under the random-effect method. The circles and horizontal lines represents the pooled OR and 95% CI by omitting a certain study. The area of the circles reflects the weight (by sample size). The diamond represents the pooled OR and 95% CI by including all of studies.

In the meta-analysis, data from 2 652 non-small cell lung cancer patients including 5 175 samples were pooled with an odds ratio of 3.45 (95% CI: 2.63–4.54) in tumor tissue versus autologous controls under random-effect method (Fig. 2). The sensitivity analysis indicated that the odds ratio range from 3.28(95% CI: 2.52–4.28) to 3.57(95% CI: 2.72–4.68) by omitting a single study under the random-effect model (Fig. 3). Only very slight change of odds ratio was seen in the sensitivity analysis, which demonstrated that the pooled odds ratio was not sensitive to a single study.

### Meta-regression and Subgroup Analysis

As the significant heterogeneity was found across the studies (I^2^ = 69.8%, χ^2^ = 135.7, *P*<0.0001), the meta-regression was performed for further evaluation of the source of heterogeneity with the Knapp-Hartung modification method. We assumed the heterogeneity may arise from the control types, age of the subjects, ethnicity of the patients, histology types, smoking status, tumor stages, sample size and the methods of methylation detection. However, complete subtype data can be only obtained in the control types, ethnicity, sample size and methylation detection methods. So, the regression was carried out by including each of complete subtypes data in the covariates. In the results of the meta-regression, no source of significant heterogeneity was found in all of them except for the control type (coefficient = −0.36, *P* = 0.018, Table 2). The τ^2^ decreased from 0.48 to 0.37, which indicates 23% [(0.48–0.37)/0.48] of heterogeneity can be explained by different control types. However, the adjustment for all the other factors with complete data mentioned above reduced the residual variance across studies only by 6%, which indicates that different ethnicity, sample size and methylation detection methods can explain only a slight proportion of the heterogeneity among studies. But for conservative, we still performed subgroup analysis according to the potential heterogeneity sources. In the subgroup analysis, the significant odds of the *P^16INK4A^* promoter methylation in tumor tissue was only changed in non-smokers (OR = 4.53, 95% CI: 0.68–30.26, *P* = 0.120) and sputum autologous control (OR = 1.49, 95% CI: 0.86–2.57, *P* = 0.151, Table 3). However, the changed of results should be interpreted with caution as only a small subject was included in non-smokers and sputum control subgroup analysis (Table 3).

**Table 2:** Meta-regression analysis.

Heterogeneity sources	Coef.(95%CI)	t	p	τ^2^	I^2^ Res(%)	R^2^(%) Adjusted
Control type	−0.36(−0.65,0.063)	−2.4	0.018	0.37	63.77	17.67
Ethnicity	0.35(−0.31,1.02)	1.07	0.29	0.45	67.72	1.06
Sample size	−0.0036(−0.011,0.004)	−0.96	0.34	0.48	68.83	−5.23
Method	−0.12(−0.61,0.38)	−0.47	0.64	0.48	68.84	−6.17

**Table 3:** Subgroup analysis.

	NSCLC		Control				
Subgroup	M+	Total	M+	Total	OR	95% CI	*p*
Sex							
Male	151	331	58	331	5.72	2.50–13.10	0
Female	34	88	11	88	5.74	2.41–13.70	0
Race							
Asia-pacific	972	2028	488	1903	3.23	2.37–4.40	0
Caucasus	293	624	151	620	4.32	2.37–7.87	0
Histology							
Sq	228	348	151	332	2.81	1.96–4.05	0
Ad	224	421	140	421	2.53	1.85–3.44	0
Other NSCLC	19	44	7	43	4.97	1.57–15.76	0.006
Smoking status							
Nonsmoker	6	32	2	32	4.53	0.68–30.26	0.12
Smoker	84	220	24	209	7.28	3.89–13.62	0
Stage							
Early (I–II)	137	405	45	394	4.62	2.29–9.30	0
Late (III–IV)	118	222	48	228	5.19	3.28–8.23	0
Method							
MSP	1114	2294	569	2166	3.49	2.58–4.70	0
RT-MSP	70	142	25	142	5.58	1.64–18.94	0.006
q-MSP	81	216	45	216	2.44	1.07–5.54	0.033
Control type							
Normal lung tissue	555	1363	155	1287	5.49	3.77–8.00	0
Blood	441	823	300	819	2.56	1.71–3.84	0
Sputum	205	357	126	287	1.49	0.86–2.57	0.151
BALF	64	109	58	130	2.97	1.16–7.65	0.024

### Correlation of *P^16INK4A^* Gene Promoter Methylation between Tumor Tissue and Autologous Clinical Samples

**Figure 4:**
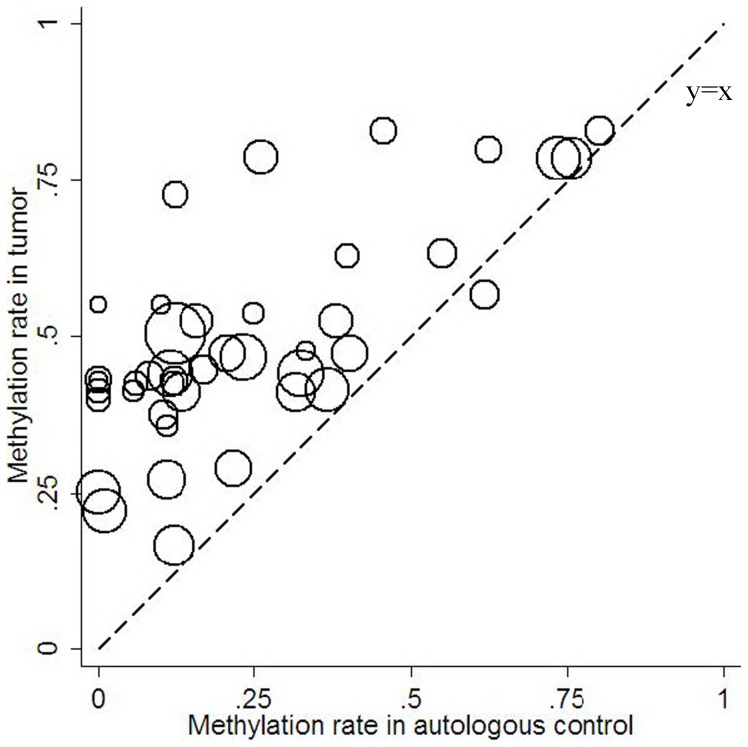
Methylation frequency in tumor tissue versus autologous controls.

**Figure 5:**
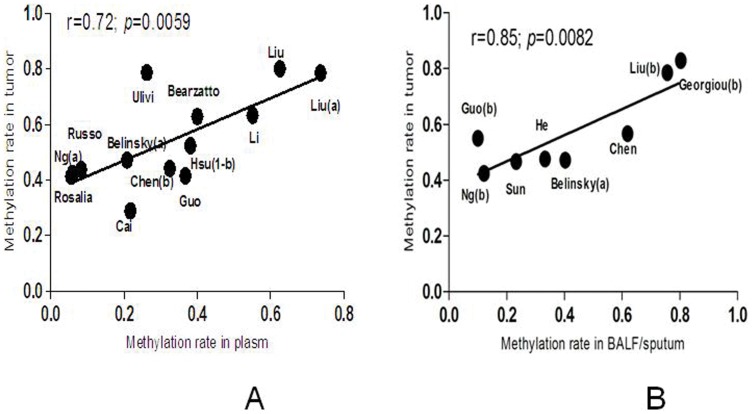
Correlation of *P^16INK4A^* promoter methylation between tumor tissue and autolougs clinical samples (A:plasm; B:BALF/sputum).

Generally, the frequency of *P^16INK4A^* promoter methylation ranged from 17% to 80% (median 44%) in the lung cancer tissue and 0 to 80% (median 15%) in the autologous controls according to the included studies. The methylation frequency in cancer tissue was much higher than that in clinical controls. We also find a strong and significant correlation between tumor tissue and autologous samples of *P^16INK4A^* promoter methylation across studies (Correlation coefficient 0.71, 95% CI:0.51–0.83, *P*<0.0001,). Fig. 4 demonstrates that most studies lie above the equal line between tumor tissue and controls, which illustrates the tumor tissue excess. In plasma samples, the methylation frequency ranged from 6% to 74% (median 33%), which showed a significant correlation of *P^16INK4A^* promoter methylation with cancer tissue (Correlation coefficient 0.72, 95% CI: 0.27–0.91, *P* = 0.0059, Fig 5A). The similar correlation was also found between the cancer tissue and sputum/BALF (Correlation coefficient 0.85, 95% CI: 0.35–0.97, *P* = 0.0082, Fig. 5B). The strong and significant correlation between tumor tissue and clinical autologous controls indicated that detection of methylation status in the clinical samples such as plasma, sputum or BALF can be a potential method for diagnosis of NSCLC without invasion.

### Publication Bias

**Figure 6:**
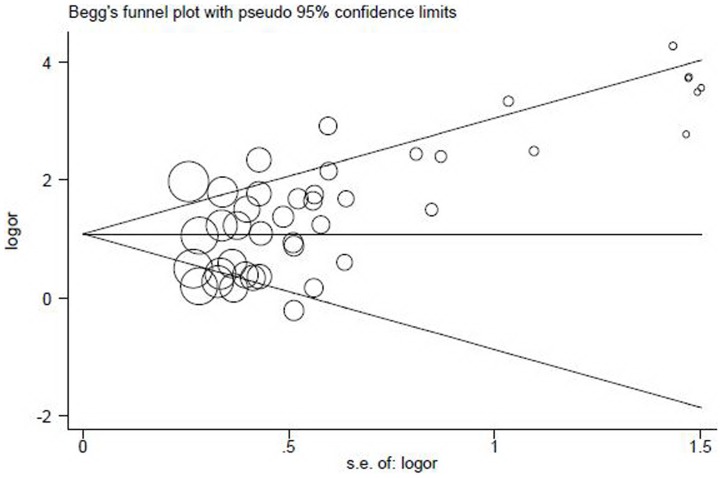
Begg’s funnel plot for assessment of publication bias. Each hollow circle represents a separate study for the indicated association. The area of the hollow circle reflects the weight (inverse of the variance). Horizontal line stands for the mean magnitude of the effect.

A Begg’s funnel plot and Egger’s test were used to evaluate possible publication bias [13]. As demonstrated in Fig. 6, the shape of the funnel plot showed a slight asymmetry at the bottom, with a trend towards reporting bigger odds ratio. However, Egger’s test did not illustrate any evidence of statistical publication bias (t = 0.78, *P* = 0.44).

## Discussion

Hypermethylation of CpG inlsnds in promoter regions is one of the important mechanisms for inactivation of tumor-suppressor genes, involving apoptosis, cell cycle, DNA repair and etc. Deregulation of the cell cycle control system was considered important in the procedure of tumorigenesis. *P^16INK4^* is known as one the most important tumor suppressor genes, which plays an important role in regulating the cell cycle. This gene generates several transcript variants that regulate the G1-S transition of the cell cycle [44]. In NSCLC, this gene product has been shown to be absence in about 32–70% of the cancer cells [45], [46]. However, mutations of the *P^16INK4^* gene are only found to be 0–10% [25], which indicating at least 22%–60% loss expression of *P^16INK4^* is associated with other mechanisms, including promoter hypermethylation.

In NSCLC, promoter hypermethylation of *P^16INK4a^* gene which encodes a cyclin-dependent kinase inhibitor, has been found in variety of studies with a frequency of 17% [26] to 83% [23] in the tumor tissue and 6% [29] to 80% [23] in autologous clinical samples. The frequency of aberrant methylation of this gene ranged from 6% [17] to 74% [18] in serum or plasma and 10% [27] to 80% [23] in sputum or BALF. Although many studies have reported the prevalence of *P^16INK4a^* gene methylation in NSCLC, the association between cancer tissue and autologous clinical samples was not definitive with the reasons of small sample size. Thus, a meta-analysis was performed to quantify the methylation-disease association, by pooling data from published studies, which can increase the statistical power.

In the present study, we included a total of thirty-four articles that reported data of methylation frequency in non-small cell lung carcinoma tissue and autologous samples. The frequency of *P^16INK4A^* promoter methylation ranged from 17% to 80% (median 44%) in the lung cancer tissue and 0 to 80% (median 15%) in the autologous controls, which shows a great variety of methylation rate between studies. In general, the pooled odds ratio of methylation was 3.45 (95% CI: 2.63–4.54) in tumor tissue versus autologous samples under random-effect method, indicating the *P^16INK4A^* promoter methylation plays an important role in the tumorigenesis of NSCLC.

In subgroup analysis, the methylation odds in tumor tissue ranged from 1.49(0.86–2.57) to 5.49(3.77–8.00) when comparing to different autologous sample sets (non-tumor lung tissue, plasma, sputum and BALF). The methylation odds in tumor tissue was not significant when comparing to sputum (*P* = 0.151) indicating no statistical different frequency of *P^16INK4A^* promoter methylation was observed between sputum and cancer tissue in non-small cell lung cancer patients. However, the results should be interpreted with caution as only a small subject was included in sputum control subgroup analysis. In other subgroups, the methylation odds in tumor tissue ranged from 2.53 (1.85–3.44) to 7.28(3.89–13.62) according to clinical characteristics such as sex, ethnicity, histology, smoking status and stages. And the highest odds 7.28(3.89–13.62) in tumor tissue was found in smokers, demonstrating smoking may play an important role in the methylation of *P^16INK4A^* promoter regions, which was in accordance with previous studies [47]. The lowest odds 2.53(1.85–3.44) in tumor tissue was shown in the adenocarcinoma, suggesting the influence of *P^16INK4A^* promoter methylation was reduced in this kind of histology type.

Generally, a strong and significant correlation between tumor tissue and autologous samples in *P^16INK4A^* promoter methylation was found across studies(Correlation coefficient 0.71, 95% CI: 0.51–0.83, *P*<0.0001), which suggested the higher frequency of methylation in autologous sample was found, the higher prevalence of methylation can be observed in cancer tissue in patients with NSCLC. And this indicated that detection of methylation status in autologous samples such as plasma, sputum or BALF can be a potential method for diagnosis of NSCLC without invasion. And according to Esteller [48], the detection of promoter hypermethylaiton in tumor suppressor genes had important clinical use, such as diagnostic tool, biomarker for prognosis, predictor for treatment responses and etc.

However, several limitations required consideration of this study. The first limitation is heterogeneity. In this meta-analysis a significant heterogeneity was existed between studies (I^2^ = 69.8%, χ^2^ = 135.7, *P*<0.0001). Although, the meta-regression was performed for further evaluation of the source of heterogeneity with the Knapp-Hartung modification method, complete data can only be obtained in the subtypes of control types, ethnicity, sample size and methylation detection methods. In the results of the meta-regression, only a small part of heterogeneity can be explained by different ethnicity, sample size and methylation detection methods, indicating that some other source of heterogeneity must be exist among studies. Second, although no evident of publication bias was found in this study by Egger’s test, the small number of studies and possible existence of unpublished articles are inevitable and completely ruling out this possibility in all aspects is difficult [49]. The third limitation is the co-variate analysis of methylation. Demonstrating by the previous studies, promoter hypermethylation was associated with many clinical, demographic and molecular features, such as gender, age, smoking status and ethnicity [21], [23], [28]. And methylation events themselves may also be linked and interact with each other, suggesting methylation analysis of a single gene may be far from enough [50]. Fourth, as known that the promoter methylation is correlated with the reduction of gene expression. However, only three articles included in this meta-analysis provided the *P^16^* gene expression status by using immunohistochemical analysis. The individual patient data (IPD) for the relationship between methylation status and expression of this gene was not given in the original articles. For the *P^16INK4A^* mutation, with carefully examination of the included studies, we found only two studies [13], [25] reported the *P^16INK4A^* mutation in exon 1, 2a and 2b regions. And none of the included 34 articles reported the mutation status of *P^16INK4A^* in promoter region.

In conclusion, the results of this study showed a higher prevalence of methylation in tumor tissue versus autologous samples in NSCLC patients, which demonstrate promoter methylation plays an important role in carcinogenesis. And the significant correlation between tumor tissue and clinical controls of *P^16INK4A^* gene promoter methylation indicated a promising biomarker for NSCLC diagnosis. However, significant methodological and validation issues remain to be addressed to provide the data that will enable this information to be considered for further clinical use [51].
